# Assessing the Role of Asymmetric Dimethylarginine in Endothelial Dysfunction: Insights Into Cardiovascular Risk Factors

**DOI:** 10.7759/cureus.77565

**Published:** 2025-01-16

**Authors:** Satyendra K Sonkar, Jyoti Verma, Gyanendra K Sonkar, Akash Gupta, Abhishek Singh, Pravesh Vishwakarma, Vivek Bhosale

**Affiliations:** 1 Medicine, King George's Medical University, Lucknow, IND; 2 Biochemistry, King George's Medical University, Lucknow, IND; 3 Cardiology, King George's Medical University, Lucknow, IND; 4 Clinical and Experimental Medicine Division, Central Drug Research Institute (CDRI), Lucknow, IND

**Keywords:** diabetic kidney disease, dyslipidemia, metabolic disorders, nitric oxide synthase, type 2 diabetes mellitus

## Abstract

Background and objective: Asymmetric dimethylarginine (ADMA) exacerbates endothelial dysfunction, which increases the risk of kidney and cardiovascular problems. This study aimed to investigate the association of ADMA levels in patients with type 2 diabetes mellitus (T2DM), including diabetic heart disease and diabetic kidney disease (DKD).

Materials and methods: This was a cross-sectional study conducted in a tertiary hospital, including 136 participants, categorizing them into four groups aged between 18-65 years: normal healthy controls (n=30), diabetes mellitus (DM) group with T2DM patients (n=39), diabetics with coronary artery disease (DCAD) group with T2DM patients with coronary artery disease (CAD) (n=34), and DKD group with T2DM patients with deranged kidney function (n=33). Comprehensive clinical and laboratory assessments were conducted, including ADMA levels quantified via enzyme-linked immunosorbent assay (ELISA) kits.

Results: In the study with 136 participants, 91 were male (66.9%), and averaging 55 years old, hypertension was prevalent in 34 participants (32.1%), more in the DKD group. Exercise adherence was low, especially among diabetic patients, but slightly better in those with complications. Among diabetics, 71 participants (67%) had uncontrolled blood sugar (glycosylated hemoglobin (HbA1c) > 7.0). Dyslipidemia was common in the DCAD group. The DKD group showed significantly low hemoglobin and proteinuria levels. The ADMA levels were significantly raised in diabetes and were maximum in the DCAD group, hence promising for predicting CAD, correlating with HbA1c and dyslipidemia.

Conclusion: In summary, our findings underscore the heightened cardiovascular vulnerability in diabetes, particularly in the presence of CAD and deranged kidney function. Endothelial dysfunction emerges as a critical factor, emphasizing the importance of monitoring ADMA levels, which can independently predict worse cardiovascular outcomes. Managing hyperglycemia, hypertension, and dyslipidemia is pivotal for reducing cardiovascular risks in diabetic patients. Further exploration of preventive strategies is warranted.

## Introduction

Type 2 diabetes mellitus (T2DM) presents a significant challenge to global health, with a prevalence of approximately 5.4% worldwide. However, the situation is particularly concerning in India, where the prevalence is notably higher at around 12.1% and occurs at a younger age compared to Western populations [1]. This increase in T2DM prevalence is primarily attributed to sedentary lifestyles and poor dietary habits.

Type 2 diabetes mellitus is a prevalent metabolic disorder that increases the risk of atherosclerotic cardiovascular disease (ASCVD) and diabetic kidney disease (DKD). The elevated cardiovascular mortality associated with T2DM is primarily linked to endothelial dysfunction, which promotes thrombosis and atherogenesis. Individuals with diabetes face a two- to four-fold higher likelihood of developing coronary artery disease (CAD) [2]. Additionally, approximately 30%-40% of T2DM patients experience nephropathy, contributing to a heightened risk of premature cardiovascular death within this subgroup of the population [3].

The metabolic and hemodynamic pathways triggered by hyperglycemia play a crucial role in the aforementioned conditions. Processes such as the activation of the renin-angiotensin system (RAS) due to oxidative stress, the presence of advanced glycosylation end products (AGE), and the oxidation of low-density lipoproteins collectively initiate and advance endothelial inflammation, thereby driving the development of diabetic vascular complications [4].

Endothelial dysfunction arises from numerous cardiovascular risk factors and systemic or local inflammation. Nitric oxide (NO), a significant vasoactive mediator derived from healthy vascular endothelium via the amino acid precursor L-arginine [5], is among the key endothelium-derived substances. One mechanism elucidating the onset of endothelial dysfunction involves elevated blood levels of asymmetric dimethylarginine (ADMA), an L-arginine analog that hinders NO formation. Consequently, this inhibition can compromise vascular function, heightening vulnerability to diabetic vascular complications [6].

This present study aimed to evaluate the association between ADMA levels and cardiovascular complications in patients with T2DM, including diabetics with coronary artery disease (DCAD) and DKD.

## Materials and methods

The study was a cross-sectional, case-control study conducted at King George’s Medical University, Lucknow, India, which started on 1^st^ August 2020 and ended on 31^st^ July 2021. The study population was divided into four groups: a normal healthy control group (n = 30), a DM group including T2DM patients (for ≥ 5 years) with estimated glomerular filtration rate (eGFR) ≥ 60 ml/min/1.73 m² without CAD (n = 39), a DCAD group including T2DM patients with eGFR ≥ 60 ml/min/1.73 m² with established CAD (n = 34), and a DKD group including T2DM patients with eGFR < 60 ml/min/1.73 m² without CAD (n = 33). The study was approved by the institution’s ethics committee under the reference number ECM II B-Thesis/P4. The procedure was carried out according to the Declaration of Helsinki of 1975 as revised in 2000 and the International Council for Harmonization-Good Clinical Practice (ICH-GCP).

Patients eligible for inclusion were those diagnosed with T2DM as per the criteria set by the American Diabetes Association (ADA), aged between 18 and 65 years, and provided written informed consent. The DCAD group was defined as T2DM patients with a history of atherosclerotic cardiovascular disease (ASCVD). Clinical signs such as chest pain, palpitations, breathlessness, or syncope, along with positive findings on either electrocardiogram (ECG) or 2D echocardiography, were indicative of CAD. The CAD diagnosis included criteria such as left ventricular ejection fraction (LVEF) ≤ 50%, left ventricular hypertrophy, left ventricular diastolic dysfunction, regional wall motion abnormality, and ECG changes indicative of previous CAD. The eGFR was determined using the Modification of Diet in Renal Disease formula (MDRD), incorporating variables such as serum creatinine, age, gender, and ethnicity [7].

eGFR = 175 x (Serum Creatinine)-1.154 x (Age)-0.203 x (0.742 only if female) x (1.212 only if Black patient)

Exclusion criteria encompassed patients with acute-on-chronic kidney disease, polycystic kidney disease, valvular heart disease, non-atherosclerotic cardiomyopathy, congenital heart disease, malignancy, inflammatory conditions, and sepsis.

A thorough examination of both clinical and laboratory data across four distinct groups was done. Detailed demographic and clinical information was meticulously documented. Plasma and serum samples were collected, centrifuged, and stored at -70°C until subsequent laboratory analysis. Using a fully automated analyzer, a wide array of tests, encompassing complete blood count (CBC), electrolyte levels (sodium and potassium), calcium (both ionic and total), markers of liver function, serum protein and albumin levels, urea/creatinine ratio, glycosylated hemoglobin (HbA1c), random blood sugar (RBS), serum cholesterol (CHL), triglycerides (TG), low-density lipoprotein (LDL), very low-density lipoprotein (VLDL), high-density lipoprotein (HDL), serum phosphate (PO43-), and uric acid (UA), were done. The levels of ADMA were quantified using commercially available enzyme-linked immunosorbent assay (ELISA) kits (Cloud Clone Corp SEA050Hu, Houston, TX, USA). Furthermore, a standard urine examination was conducted to determine the albumin-creatinine ratio (UACR), expressed as mg albumin/g creatinine.

Statistical analysis

Baseline characteristics were evaluated using standard descriptive statistics. Continuous variables were expressed as mean ± standard deviation, while categorical variables were presented as counts and percentages. Group-wise comparisons for quantitative variables were conducted using an F-test, whereas qualitative variables were compared using either the chi-square test or Fisher's exact test as appropriate. A significance threshold of p < 0.05 was adopted. The one-way analysis of variance (ANOVA) was utilized to ascertain significant differences between the means of multiple independent groups. The strength of linear associations between variables was measured using the Pearson correlation coefficient, where a value of r = 1 indicates a perfect positive correlation and r = -1 indicates a perfect negative correlation. Data were entered into a Microsoft Excel spreadsheet (Microsoft Corp., Redmond, WA, USA) and analyzed using IBM SPSS Statistics software version 29.0 (IBM Corp., Armonk, NY, USA).

## Results

The study comprised 136 participants, with 91 (66.9%) males and 45 (33.1%) females. Among these, 30 were healthy controls, and 106 were patients with T2DM, including those with diabetic complications (DCAD and DKD) and those without (DM group). The majority of diabetic patients fell within the 50-60 years of age group, with a mean age of 55±7.6 years. Hypertension was the most prevalent comorbidity, affecting 32.1% of diabetic patients, particularly prominent in the DKD group. Only a quarter of DM patients adhered to exercise, whereas adherence was higher (about 50%) among those with complications (DCAD and DKD groups). Patients with complications exhibited significantly improved healthy lifestyle practices (81.8% and 88.3% in DKD and DCAD, respectively, versus 61.6% in DM), possibly indicating a lack of awareness until complications arise. Baseline comparisons of various parameters are summarized in Table 1.

**Table 1:** Baseline comparison of groups based on biochemical parameters BMI: body mass index; Hb: hemoglobin; TLC: total leucocyte count; MCV: mean corpuscular volume; MCH: mean corpuscular hemoglobin; RBS: random blood sugar; ACR: albumin creatinine ratio; Na: sodium; K: potassium; eGFR: estimated glomerular filtration rate; S. Bil (T): serum bilirubin total; S. Bil (D): serum bilirubin direct; Ca: calcium; Mg: magnesium; PO4: phosphate; HDL: high-density lipoprotein; LDL: low-density lipoprotein; VLDL: very low-density lipoprotein; HbA1c: glycosylated hemoglobin; ADMA: asymmetrical dimethylarginine

Parameter	Reference range (unit)	Control (n=30)		T2DM (n=39)		DKD (n=33)		DCAD (n=34)		ANOVA	
		Mean	SD	Mean	SD	Mean	SD	Mean	SD	F-value	P-value
Age	18-65 (years)	45.3	5.6	52.9	8.2	56.8	6.0	55.7	7.9	2.5	0.08
BMI	18.5-24.9 (kg/m^2^)	24.9	3.4	24.5	4.3	23.5	5.1	26.0	5.4	2.0	0.14
Hb	12-15 (gm/dL)	13.2	0.8	10.6	1.9	9.5	1.8	11.4	2.1	23.2	<0.001
TLC	4000-10,000 (per mm^3^)	8492	2167	9795	3755	9272	3585	10297	2575	1.9	0.13
MCV	80-100 (fL)	83.2	6.2	81.8	7.9	84.0	5.3	82.6	6.3	0.7	0.55
Platelet	1.5-4.5 (10^ 5^/µL)	2.2	0.8	2.0	0.9	1.6	0.6	1.9	0.8	3.2	0.02
RBS	70-140 (mg/dL)	90.4	16.0	210.9	78.7	237.9	105.2	202.0	91.8	19.8	<0.001
Urine ACR	<30 (mg/gm)	17.6	4.6	61.3	75.9	507.2	753.0	100.9	152.4	11.6	<0.001
Na	135-145 (mEq/L)	138.8	5.3	131.8	6.3	136.2	9.1	133.1	8.6	5.8	0.001
K	3.5-4.5 (mEq/L)	4.3	0.7	3.8	0.8	4.3	1.0	5.3	5.6	1.6	0.17
Urea	12.9-42.9 (mg/dL)	39.0	7.3	43.2	31.1	112.9	70.3	43.0	32.7	23.9	<0.001
Creatinine	0.5-1.4 (mg/dL)	0.9	0.2	0.9	0.2	3.2	1.8	0.9	0.2	50.5	<0.001
eGFR	≥60 (ml/min/1.73m2)	93.6	32.4	83.0	35.1	23.6	10.6	79.2	22.3	43.8	<0.001
SGOT	<35 (IU/mL)	32.9	8.0	40.2	24.4	36.7	18.6	71.4	101.0	3.6	0.01
SGPT	<35 (IU/mL)	31.2	9.4	50.9	41.9	34.9	30.2	107.0	278.0	2.03	0.11
S.ALP	<240 (IU/mL)	193.7	47.9	255.7	155.5	281.5	237.7	241.7	166.9	1.51	0.21
S. Protein	6.0-7.8 (gm/dL)	6.5	0.5	5.8	1.0	5.7	0.9	6.0	0.9	3.8	0.01
S. Albumin	3.5-5.0 (gm/dL)	4.4	0.6	3.5	1.0	3.1	0.8	3.5	1.1	10.8	<0.001
Ca Total	8.5-10.5 (mg/dL)	8.3	1.4	6.9	1.8	6.9	1.8	6.4	1.8	6.5	<0.001
Mg	1.7-2.2 (mg/dL)	2.1	0.3	2.4	0.8	2.4	0.6	2.4	0.7	1.7	0.17
PO4	2.4-4.4 (mg/dL)	3.9	0.8	4.4	1.3	5.2	1.4	4.5	1.0	6.7	<0.001
Uric Acid	2.6-6.0 (mg/dL)	5.1	1.3	6.6	2.8	7.6	2.5	5.8	2.8	6.0	0.001
Cholesterol	<200 (mg/dL)	137.6	16.2	142.6	35.9	138.7	33.0	165.0	126.6	1.1	0.32
Triglyceride	<150 (mg/dL)	111.5	32.8	150.4	88.9	117.7	48.1	155.1	66.0	3.9	0.01
HDL	>50 (mg/dL)	46.5	13.2	52.5	20.2	54.2	22.1	42.0	11.4	3.4	0.02
LDL	<100 (mg/dL)	59.4	14.4	61.6	29.4	59.9	38.1	65.6	32.0	0.3	0.83
VLDL	<30 (mg/dL)	20.6	7.2	29.4	21.6	23.4	11.7	24.6	10.3	2.3	0.08
HbA1C	4.0-5.6 (% )	4.9	0.5	8.6	2.9	8.1	2.2	7.8	1.6	20.0	<0.001
ADMA	0-100 (ng/mL)	89.6	83.3	241.2	179.8	247.9	165.3	339.3	204.5	12.0	<0.001

The majority of diabetic patients (67%) had elevated HbA1c (≥7%), indicative of uncontrolled diabetes. Dyslipidemia was prevalent across diabetic groups, with the DCAD group showing the lowest HDL cholesterol (42.0±11.4 mg/dl) and highest TG levels (155.1±66.0 mg/dl). Platelet count was significantly decreased in diabetic patients, with the lowest value (1.6±0.6 10^ 5^/µL) observed in the DKD group. Among the subgroups (DM, DKD, and DCAD), mean BMI was highest in the DCAD group (26±5.4 kg/m²) and lowest in the DKD group (23.5±5.1 kg/m²). Significant differences were found for Hb levels (P=0.001), with the lowest levels in the DKD group (9.5±1.8 g/dl). The DKD patients exhibited significantly elevated UACR (386±273.4 mg/g), hyperuricemia (7.6±2.5 mg/dl), and hyperphosphatemia (5.3±1.5 mg/dl). Comparisons with healthy controls revealed significant differences in ADMA levels (P < 0.001), with the highest in the DCAD group (339.3 ± 204.5 ng/ml) and the lowest in controls (89.6 ± 83.3 ng/ml) (Table 1). However, ADMA levels did not significantly differ between diabetic patients with or without complications (Table 2). Receiver operating characteristic (ROC) curve analysis was conducted to evaluate the predictive ability of ADMA for significant CAD compared to the eGFR.

**Table 2:** Paired comparisons of ADMA ADMA: asymmetrical dimethylarginine; T2DM: type 2 diabetes mellitus; DKD: diabetic kidney disease; DCAD: diabetics with coronary artery disease statistical test: post hoc Tukey test

Group vs. Group		ADMA (ng/mL)		p-value
		Mean Diff. (I-J)	SE	
Control	T2DM	-151.6	40.5	0.002
Control	DKD	-158.2	42.1	0.001
Control	DCAD	-249.7	41.8	<0.001
T2DM	DKD	-6.6	39.5	0.998
T2DM	DCAD	-98.1	39.2	0.064
DKD	DCAD	-91.4	40.8	0.118

**Figure 1:**
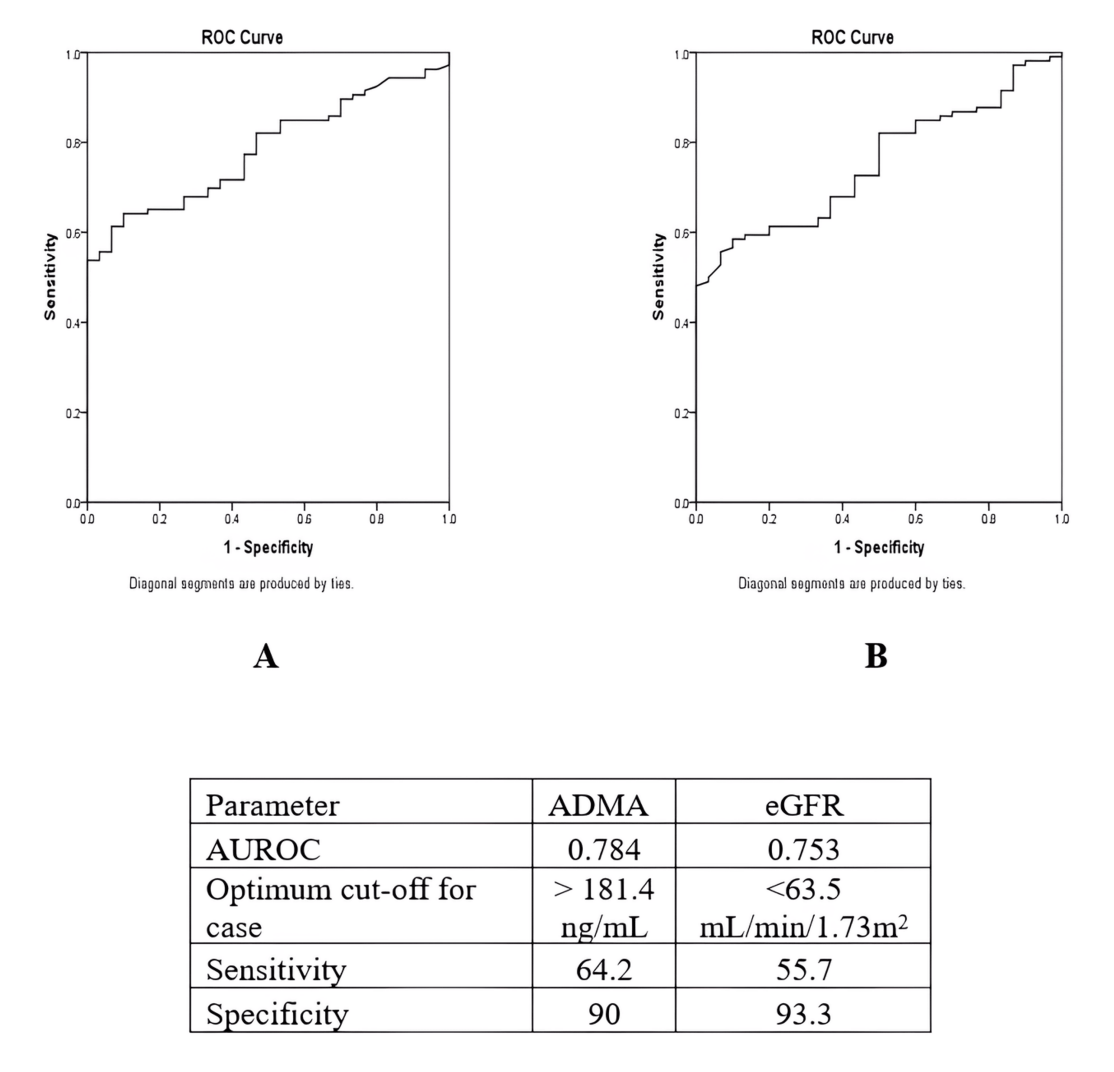
The ROC comparison between eGFR and ADMA for the separation of patients with and without significant coronary artery disease panel A: ROC curve for ADMA; panel B: ROC curve for eGFR ROC: receiver operating characteristic curve analysis; AUROC: area under the ROC curve; ADMA: asymmetrical dimethylarginine; eGFR: estimated glomerular filtration rate

The ROC curve for ADMA showed an area under the curve (AUC) indicating that ADMA is a slightly superior predictor of significant CAD. The optimal cut-off value for ADMA was identified above 181.4 ng/ml. At this threshold, the sensitivity was 64.2%, and the specificity was 90%. This suggests that ADMA can effectively differentiate between patients with and without significant CAD, with a reasonably high level of specificity and moderate sensitivity. These results highlight the potential of ADMA as a biomarker for significant CAD, outperforming eGFR in this context (Figure 1).

The ADMA correlated significantly with HbA1c (r=0.21, P<0.05) and triglyceride levels (r=0.19, P<.05), suggesting associations with uncontrolled diabetes and dyslipidemia (Table 3).

**Table 3:** Linear correlation between ADMA and other variables ADMA: asymmetrical dimethyl arginine; BMI: body mass index; ACR: albumin-creatinine ratio; LDL: low-density lipoprotein; VLDL: very low-density lipoprotein; HDL: high-density lipoprotein

ADMA	Age	BMI	Urine ACR	HbA1c	Cholesterol	Triglyceride	LDL	VLDL	HDL
Pearson correlation (r)	-0.10	0.07	-0.04	0.21	- 0.007	0.19	0.006	0.13	-0.05
P-value	0.27	0.93	0.68	<0.05	0.93	< 0.05	0.95	0.13	0.57

## Discussion

There was an increased prevalence of diabetes mellitus at a younger age compared to the Western population, and also the complication occurred a decade earlier. This could be attributed to the Asian Indian phenotype characterized by low BMI, higher body fat, and waist circumference suggestive of increased visceral adiposity, which plays a key role in an inflammatory cascade [3]. People who have diabetes are more likely to develop ASCVD. Moreover, renal impairment accelerates the development of arteriosclerosis and atherosclerosis, which increases the risk of premature mortality [2]. Chronic inflammation and endothelial dysfunction are the main causes of this increased risk of dying from heart disease. Heart disease is caused by known risk factors such as a sedentary lifestyle, smoking, obesity, diabetes, hypertension, and proteinuria. These ultimately point to endothelial dysfunction as the primary mechanism responsible for cardiovascular problems. This dysfunction is mainly brought on by impaired NO production.

**Figure 2:**
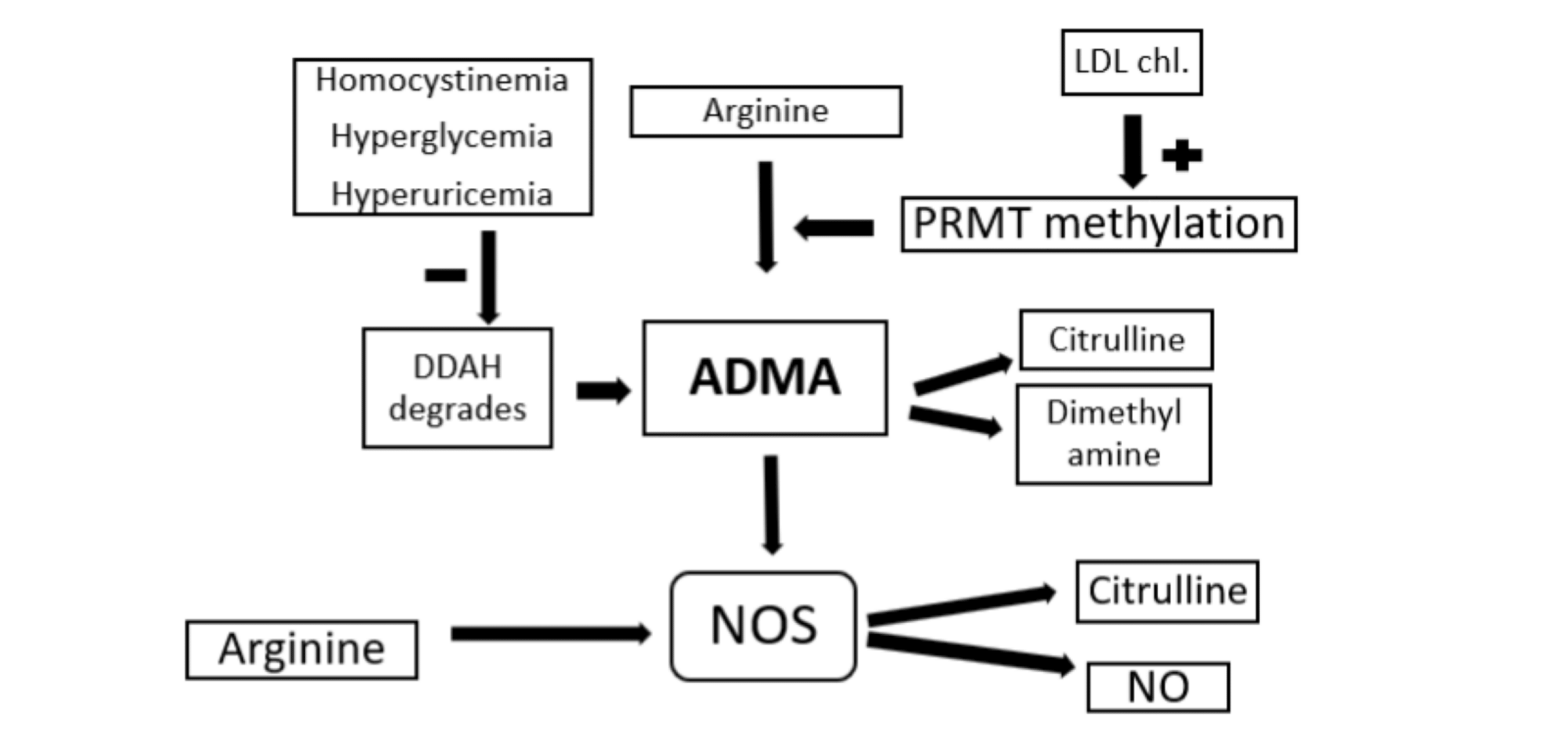
Mechanism of action of ADMA ADMA: asymmetric dimethylarginine; DDAH: dimethylarginine dimethylaminohydrolase; PRMT: protein arginine methyl transferase; NOS: nitric oxide synthase; NO: nitric oxide; LDL Chl: low-density lipoprotein cholesterol Image credits: Dr. S K Sonkar

Endothelial dysfunction, which results from reduced NO generation and availability, is thought to be the first step in the process [5]. Nitric oxide is a well-known anti-atherogenic substance that inhibits atherosclerosis's proliferative and inflammatory processes. Nitric oxide synthase (NOS), activated by L-arginine, converts L-arginine into NO. However, endogenous L-arginine analogs such as ADMA can suppress this process. The naturally occurring amino acid ADMA is found in plasma and other organs. It is produced when proteins with methylated arginine residues break down [5,6]. Asymmetric dimethylarginine inhibits NOS catalytic activity, which limits NO release [8]. It was from human urine that the endogenous inhibitor of NOS, ADMA, was first identified and purified in 1970. The enzyme protein arginine methyl transferase (PRMT) facilitates the dimethylation of L-arginine inside protein residues, which produces ADMA. Primarily, dimethylarginine dimethylaminohydrolase (DDAH) breaks it down through competitive interaction with arginine; ADMA inhibits NOS (Figure 2) [9]. However, in diabetic individuals, heightened levels of PRMT expression and diminished DDAH activity, attributable to hyperglycemia and dyslipidemia-induced reactive oxygen species (ROS) production, lead to ADMA accumulation [8,10]. At the molecular level, elevated LDL-C levels contribute to endothelial cell dysfunction, reducing NO bioavailability through compromised L-arginine transport and metabolism and uncoupling of endothelial NOS (eNOS) [11].

Increased levels of ADMA are linked with conditions including atherosclerosis, hypertension, chronic heart failure (CHF), DM, and chronic renal failure (CRF) [12]. We found that the DM, DKD, and DCAD groups had significantly higher levels of ADMA. Elevated ADMA levels outperform conventional risk variables as the most powerful predictor of cardiovascular events and mortality. Endothelial dysfunction worsens with age and is correlated with an increase in ADMA (r=0.2, P<0.01), which is caused by the progression of vascular atherosclerosis [11,12]. However, the early elevation of ADMA brought on by the illness condition and its longevity are the main topics of this discussion. Both BMI and ADMA levels were higher in diabetic patients with CAD. The relationship between lipotoxicity and insulin resistance and obesity, a known risk factor for both diabetes and cardiovascular illnesses, helps to explain this association. Our results are supported by Krzyzanowska et al., who showed a substantial rise (P=0.04) in ADMA levels in obese persons relative to normal-weight individuals [13]. High TG, low levels of HDL-C, and elevated LDL-C are the usual symptoms of diabetic dyslipidemia [14]. Significant differences were found in the levels of TG and HDL-C in patients with sequelae from diabetes. Increased ADMA levels and dyslipidemia have been linked in previous studies [13,15]. Despite having higher ADMA levels, the DKD group had the lowest BMI in contrast to the previously described data [16]. The sarcopenia can explain this disparity that patients with chronic kidney disease endure, which lowers lean body mass. Along with increasing ADMA levels, another factor that may contribute to these phenomena in CKD patients is the increased prevalence of atherosclerosis. These results were supported by another investigation, which showed that although CKD patients’ mean BMI was lower (21.6±3.5 kg/m²) than that of healthy controls (22.4±2.9 kg/m²), they had considerably higher levels of ADMA [17].

The ADMA levels of patients with albuminuria were higher than those of patients without albuminuria, but the difference was not statistically significant. Notably, those with albuminuria have an increased risk of CVD. Previous studies have consistently shown that proteinuria and ADMA have a substantial positive connection. In particular, research has shown that the macroalbuminuria group had far greater levels of ADMA than the microalbuminuria group [6,18]. Chronic inflammation is caused by uremic toxins in DKD patients, which can lead to problems such as anemia, hypertension, and mineral bone disease. When taken as a whole, these variables increase the risk of CVD and death. Atherosclerotic cardiovascular disease is greatly increased by hypertension, and diabetic patients have twice the chance of developing hypertension in comparison to the general population. Diabetic kidney disease develops in about 30%-40% of diabetic people; DKD frequently coexists with hypertension [19]. Studies show that compared to individuals with diabetes or hypertension alone, ADMA levels are much higher in diabetic patients with hypertension. Elevated levels of ADMA are a result of increased endothelial shear and stress caused by hypertension, and this is especially true in patients with essential hypertension [5,18].

The DKD group exhibited the lowest hemoglobin levels, with a mean of 9.5±1.8 g/dL. Anemia in DKD is predominantly attributed to deficiencies in iron and/or erythropoietin [20]. Additionally, gut edema resulting in malnutrition secondary to heart failure and elevated inflammatory cytokines are believed to contribute to anemia in patients with CAD [21]. Secondary hyperparathyroidism leading to bone marrow fibrosis is another significant factor. Mineral bone disorders are implicated in advanced atherosclerosis, where an increased calcium-phosphate product precipitates progressive calcium deposition in the coronary arteries, as well as the mitral and aortic valves, particularly in patients with advanced renal failure [22]. High blood phosphate levels are associated with a considerable risk of CVD and death in both the general population and people with CKD. Hypertension, atherosclerosis, left ventricular hypertrophy, cardiac valve calcification, and myocardial fibrosis are all influenced by elevated phosphate levels [22]. We found that the DKD and DCAD groups had higher serum phosphate levels during our investigation. The DKD group showed a significant rise in uric acid levels, which is typical of hyperuricemia brought on by renal failure. Significant risk factors for atherosclerosis, such as insulin resistance, metabolic syndrome, hypertension, and renal failure, are intimately associated with hyperuricemia. Hyperuricemia-induced endothelial dysfunction may result from increased levels of ADMA due to NOX/ROS signaling pathway activation and decreased DDAH activity, and previous research in CKD patients has demonstrated that both elevated uric acid and ADMA levels are associated with an increased risk of cardiovascular events [23].

In our investigation, we observed the highest ADMA levels in DCAD. Nonetheless, our study did not detect statistically significant inter-group differences among diabetic patients without complications, those with DKD, and those with DCAD. Therefore, it suggests that all diabetic patients may be at an increased risk of CAD.

In our research, we observed a notable correlation between plasma ADMA levels and HbA1c concentration, indicating that diabetic patients with poor metabolic control face an elevated risk of CAD. This suggests that compromised metabolic control heightens the risks of endothelial dysfunction and vasculopathy [3]. Previous studies have similarly demonstrated a significant correlation between plasma ADMA levels and HbA1c in diabetic patients [24,25]. However, research by Karakoc et al. and Hsu et al. found no such correlation between ADMA and HbA1c [26,27].

Drugs that address dyslipidemia, hypertension, and hyperglycemia can affect ADMA levels, which will ultimately enhance endothelial function. Certain medications are effective in lowering ADMA levels, including angiotensin-converting enzyme (ACE) inhibitors, ezetimibe, statins, sodium-glucose cotransporter-2 (SGLT2) inhibitors, thiazolidinediones, and metformin [15,28]. Increased ADMA levels are linked to the beginning of menopause and estrogen's promotion of NO generation. Studies show that hormone replacement treatment (HRT) can lower ADMA levels and may even have positive effects. Furthermore, it has been demonstrated that supplementing with L-arginine, a direct precursor of NO, improves endothelial function and lowers ADMA levels [29,30].

Limitations

A large, multicentric, prospective study is needed to validate the findings of the present study. A standardized value of ADMA would be required for using it as a universal biomarker for endothelial dysfunction. Drugs can modify the level of ADMA, but detailed drug supplementation was not considered here. Dietary supplementation of L-arginine would also affect the level of ADMA, and it would be interesting to know its role in the future progression of the disease and its complications.

## Conclusions

Our research highlights the increased risk of CVD in people with diabetes, especially when it comes to comorbidities like DCAD and DKD. One key mechanism that emerges is endothelial dysfunction, which is caused by a variety of variables such as chronic inflammation and decreased generation of NO. Our results demonstrate the therapeutic potential of focusing on the levels of ADMA, which are important in endothelial dysfunction. In addition to ADMA regulation, medications and therapies targeting hyperglycemia, hypertension, and dyslipidemia show promise in reducing the risk of CVD in diabetics. It is necessary to conduct additional research to investigate new preventive measures.
